# Rapamycin toxicity in MIN6 cells and rat and human islets is mediated by the inhibition of mTOR complex 2 (mTORC2)

**DOI:** 10.1007/s00125-012-2475-7

**Published:** 2012-02-08

**Authors:** A. D. Barlow, J. Xie, C. E. Moore, S. C. Campbell, J. A. M. Shaw, M. L. Nicholson, T. P. Herbert

**Affiliations:** 1 Department of Cell Physiology and Pharmacology, University of Leicester, The Henry Wellcome Building, University Road, Leicester, LE1 9HN UK; 2 Transplant Surgery Group, Department of Infection, Immunity and Inflammation, University of Leicester, Leicester, UK; 3 Institute of Cellular Medicine, Newcastle University, Newcastle, UK

**Keywords:** Apoptosis, Beta cell, Diabetes mellitus, GSIS, Islet, Islet transplantation, mTOR, mTORC2, PKB, Rapamycin, RICTOR

## Abstract

**Aims/hypothesis:**

Rapamycin (sirolimus) is one of the primary immunosuppressants for islet transplantation. Yet there is evidence that the long-term treatment of islet-transplant patients with rapamycin may be responsible for subsequent loss of islet graft function and viability. Therefore, the primary objective of this study was to elucidate the molecular mechanism of rapamycin toxicity in beta cells.

**Methods:**

Experiments were performed on isolated rat and human islets of Langerhans and MIN6 cells. The effects of rapamycin and the roles of mammalian target of rapamycin complex 2 (mTORC2)/protein kinase B (PKB) on beta cell signalling, function and viability were investigated using cell viability assays, insulin ELISA assays, kinase assays, western blotting, pharmacological inhibitors, small interfering (si)RNA and through the overproduction of a constitutively active mutant of PKB.

**Results:**

Rapamycin treatment of MIN6 cells and islets of Langerhans resulted in a loss of cell function and viability. Although rapamycin acutely inhibited mTOR complex 1 (mTORC1), the toxic effects of rapamycin were more closely correlated to the dissociation and inactivation of mTORC2 and the inhibition of PKB. Indeed, the overproduction of constitutively active PKB protected islets from rapamycin toxicity whereas the inhibition of PKB led to a loss of cell viability. Moreover, the selective inactivation of mTORC2 using siRNA directed towards rapamycin-insensitive companion of target of rapamycin (RICTOR), mimicked the toxic effects of chronic rapamycin treatment.

**Conclusions/interpretation:**

This report provides evidence that rapamycin toxicity is mediated by the inactivation of mTORC2 and the inhibition of PKB and thus reveals the molecular basis of rapamycin toxicity and the essential role of mTORC2 in maintaining beta cell function and survival.

**Electronic supplementary material:**

The online version of this article (doi:10.1007/s00125-012-2475-7) contains peer-reviewed but unedited supplementary material, which is available to authorised users.

## Introduction

Since the publication of the landmark Edmonton study in 2000 [1], use of rapamycin (sirolimus) has been at the forefront of immunosuppression for islet transplantation. The employment of rapamycin as the primary immunosuppressant in the Edmonton protocol allowed the avoidance of glucocorticoids and minimisation of calcineurin inhibitors, both known to be profoundly diabetogenic [1].

Although the initial results of the Edmonton study were very promising, enthusiasm was tempered when the 5 year results of the initial cohort of patients were reported, with only approximately 10% of recipients maintaining insulin independence [2]. Although the cause of loss of graft function/viability is poorly understood there is growing evidence that it is, in part, due to rapamycin toxicity [3–5].

Rapamycin has been shown to have detrimental effects on the function and survival of murine pancreatic beta cell lines [4], cultured murine islets [3, 5] and cultured human islets [4] as well as of a mouse syngeneic islet-transplant model [5]. In addition, postmortem examination of a patient with a failed islet graft transplanted under the Edmonton protocol showed no evidence of autoimmune or alloimmune damage to the transplanted islets, suggesting the failure was due to non-immunological causes, including drug toxicity [6].

Rapamycin exerts its pharmacological actions via inhibition of the serine/threonine kinase mammalian target of rapamycin (mTOR) [7]. mTOR exists in two complexes, mTOR complex 1 (mTORC1) and mTOR complex 2 (mTORC2). Both mTOR complexes contain mTOR, mammalian orthologue of lethal with sec thirteen (mLST8), DEP (dishevelled, egl-10, pleckstrin) domain-containing mTOR interacting protein (DEPTOR), the newly discovered 58 kDa glucose-regulated protein (GRP58), Tel2 interacting protein 1 (TTI1), telomere maintenance 2 (TEL2) and Ras (rat sarcoma)-related C3 botulinum toxin substrate 1 (RAC1) [8]. In addition, mTORC1 contains regulatory-associated protein of target of rapamycin (RAPTOR) and pro-rich Akt substrate of 40 kDa (PRAS40), while mTORC2 contains rapamycin-insensitive companion of TOR (RICTOR), mammalian stress activated protein kinase interacting protein 1 (mSIN1) and protein observed with RICTOR (PROTOR) [7, 8]. mTORC1 is highly sensitive to rapamycin whereas, in general, mTORC2 is rapamycin insensitive [9]. However, it has been shown in some cell types that prolonged rapamycin treatment inhibits mTORC2 assembly [10, 11].

mTORC1 is activated by nutrients, growth factors and cellular energy levels [7] and plays a key role in the regulation of beta cell size and proliferation [8]. Indeed, beta cell specific tuberous sclerosis complex-2 (TSC2; an upstream negative regulator of mTORC1 [12]) knockout mice have increased beta cell mass due to increased cell size and proliferation [13, 14]. The effects of mTORC1 on cell size are likely to be mediated by the activation of ribosomal S6 protein kinase (S6K) 1 and 2, downstream targets of mTORC1, as *S6k1* (also known as *Rps6kb1*)-knockout mice [15] and *Rps6*-knockin mice, with non-phosphorylatable ribosomal protein S6 (RPS6) [16] have comparatively smaller beta cells than wild-type mice.

mTORC2 was originally identified as a mediator of actin cytoskeletal organisation, polarisation and cell migration [9], and is responsible for the phosphorylation and activation of several members of the AGC kinase subfamily, including protein kinase B (PKB, otherwise known as AKT), serum/glucocorticoid-induced kinase 1 (SGK1), conventional protein kinase Cs (PKCs) and PKCε [7]. Recently, it has been reported that beta cell-specific deletion of *Rictor* in mice (i.e. beta cell-specific *Rictor* knockout mice [βRicKO]) results in a reduction in beta cell mass (due to impaired proliferation but not changes in cell size or cell death) accompanied by moderate hyperglycaemia and glucose intolerance [17].

The initial objective of this study was to elucidate the molecular basis for rapamycin toxicity in islets. This led to the discovery that rapamycin treatment of beta cells not only inhibits mTORC1 but also inhibits mTORC2. More importantly, we provide evidence that the molecular basis of rapamycin toxicity is through the inactivation of mTORC2 and its impact on PKB activity. These results reveal a hitherto unknown essential role for mTORC2 in maintaining beta cell function and viability.

## Methods

### Reagents

Unless otherwise stated, all chemicals and reagents were purchased from Sigma-Aldrich (St Louis, MO, USA). FCS was purchased from Invitrogen (Carlsbad, CA, USA). [γ^32^P]ATP was purchased from GE Healthcare (Piscataway, NJ, USA). Rapamycin was purchased from Calbiochem (Nottingham, UK). Torin1 [18] was kindly provided by D. Sabatini (Whitehead Institute for Biomedical Research, Cambridge, MA, USA). Recombinant adenovirus producing a myristylated version (constitutively active) of PKB (AdCaPKB) was purchased from Vector Biolabs (Philadelphia, PA, USA).

### Cell culture and treatments

MIN6 cells [19] were used between passages 20 and 45 at approximately 80% confluence and grown as previously described [20]. Treatments were performed as described in the figure legends.

### Islet isolation, culture and treatment

Pancreatic islets were isolated from male Sprague–Dawley rats, weighing 200–250 g, by collagenase digestion and Histopaque density-gradient centrifugation as previously described [21]. Rat islets were cultured in RPMI 1640 containing 5.6 mmol/l glucose, 100 U/ml penicillin and 100 μg/ml streptomycin. Human islets were isolated from pancreases from heart-beating deceased human donors following ethical approval and informed consent from the donors' relatives. Islets were isolated at the Scottish National Blood Transfusion Service Islet Isolation Facility, Edinburgh, UK [22], and transported to Newcastle University in CMRL 1066 (Cellgro, Herndon, VA, USA), containing 0.5% (wt/vol.) human serum albumin and 5,000 U heparin. Human islets were cultured in CMRL-NCL1 (PAA Laboratories, Yeovil, UK) containing 1% human serum albumin, 100 U/ml penicillin and 100 μg/ml streptomycin, prior to experimentation. Following treatment, rat and human islets were collected by centrifugation for 1 min at 200 *g* and lysed in ice-cold lysis buffer.

### SDS-PAGE and western blotting

SDS-PAGE and western blotting were performed as described previously [20]. Anti-mTOR, anti-RAPTOR, anti-RICTOR, anti-PKB, anti-cleaved caspase 3, anti-RPS6, anti-S6K1, anti-phosphorylated (P)-PKB Ser473, anti-P-PKB Thr308, anti-P-RPS6 Ser240/244, anti-P-S6K1 Thr389, anti-P-forkhead box O (FOXO)1/FOXO3a Thr24/Thr32, anti-P-glycogen synthetase kinase 3 (GSK3)α/β Ser21/9 and anti-P-PKCα Thr638/641 antibodies used for western blotting were purchased from Cell Signalling Technologies (Beverly, MA, USA). Anti-glyceraldehyde-3-phosphate dehydrogenase (GAPDH) was purchased from Santa Cruz Biotechnology (Santa Cruz, CA, USA). Anti-PKCα was purchased from Transduction Laboratories (Oxford, UK). Anti-mTOR and anti-RICTOR antibodies used for immunoprecipitation were purchased from the Division of Signal Transduction Therapy, University of Dundee, UK.

### Infection of cell lines with recombinant adenoviruses

Adenovirus-mediated transduction of cell lines was performed as previously described [23].

### Immunoprecipitation of mTOR and RICTOR

For immunoprecipitation, MIN6 cells were lysed in 3-[(3-cholamidopropyl)dimethylammonio]-1-propanesulfonate hydrate (CHAPS) lysis buffer and the mTOR complexes were isolated essentially as described previously [24].

### Glucose-stimulated insulin secretion assay

Following treatment, islets or MIN6 cells were incubated in KRB supplemented with 1 mmol/l glucose for 60 min at 37°C. The supernatant fractions were collected and the incubation continued in KRB containing 20 mmol/l glucose for a further 60 min at 37°C. The supernatant fractions were again collected. For MIN6 cells, the cell pellets were lysed in ice-cold acid/ethanol solution (HCl 1.5% [vol./vol.], ethanol 75% [vol./vol.] and H_2_O 23.5% [vol./vol.]) prior to measurement of cellular insulin content. Insulin concentration in the supernatant fractions or pellets were assayed using an anti-mouse (for MIN6 cells) or anti-rat (for rat islets) insulin ELISA kit (DRG Instruments, Marburg, Germany) with mouse or rat insulin as a standard in accordance with the manufacturer’s instructions. The absorbance was read at 450 nm on a Novostar plate reader (BMG Labtech, Cary, NC, USA).

### PKB kinase assay

MIN6 cells were infected with a recombinant adenovirus producing constitutively active PKB as described above. At 24 h post infection, MIN6 cells were treated and lysed as described in the figure legends. PKB was immunoprecipitated from the lysates using anti-PKB antibody (Millipore, Watford, UK) as per the manufacturer’s instructions and the activity of PKB determined using Crosstide as a substrate peptide (GRPRTSSFAEG; 30 mmol/l; Millipore) as previously described [25].

### Annexin V/propidium iodide staining

Following treatment, the media were removed and kept. The cells were then incubated in ×1 trypsin/EDTA (0.5%) for 4 min at 37°C. DMEM was added and the cells gently dispersed by pipetting, combined with the saved media and centrifuged at 200 *g* for 5 min at room temperature. The media were discarded and the cell pellets gently resuspended in DMEM and equilibrated by incubation at 37°C for 30 min. The cells were pelleted by centrifugation at 200 *g* for 10 min at room temperature and the media removed. Annexin V binding and propidium iodide staining were performed using the Annexin-V-Fluos staining kit (Roche, Burgess Hill, UK) as per the manufacturer’s instructions. Quantification of staining was performed using a FACScan or FACSCalibur flow cytometer, and CellQuest software (BD Biosciences, San Jose, CA, USA).

### Cell death detection assay

Following islet culture and treatment, evaluation of cell death was performed using the Cell Death Detection ELISA^PLUS^ kit (Roche, Burgess Hill, UK), as per the manufacturer’s instructions. Absorbance was measured at 405 nm against 2,2′-azino-bis(3-ethylbenzothiazoline-6-sulphonic acid) (ABTS) solution and ABTS stop solution as a blank using a Novostar plate reader (BMG Labtech, Aylesbury, UK) and the results expressed in arbitrary units of oligonucleosome-associated histone.

### Small interfering RNA transfection of dispersed islets

Islets were dispersed essentially as described by Jonkers et al. [26]. Small interfering (si)RNA transfection was performed using Lipofectamine 2000 (Invitrogen, Carlsbad, CA, USA) according to the manufacturer’s instructions. For *Rictor* or *Raptor* (also known as *Rptor*) knockdown, the cells were transfected for 48 h with 200 nmol/l of on-target plus SMARTpool small interfering RNA (siRNA) against *Rictor* (L-087724-00-0005) or *Raptor* (L-086862-00-0005), respectively. siGENOME non-targeting siRNA (Dharmacon, Epsom, UK; scrambled), 200 nmol/l, was used as a control.

### Quantification and statistical analysis

Immunoblot-band intensities were quantified using the ImageJ (version 1.44) software. Statistical analyses were performed as indicated in the figure legends using GraphPad Prism 5.0 (GraphPad Software, San Diego, CA, USA).

## Results

### Rapamycin has deleterious effects on MIN6 cell viability and function

**Fig. 1:**
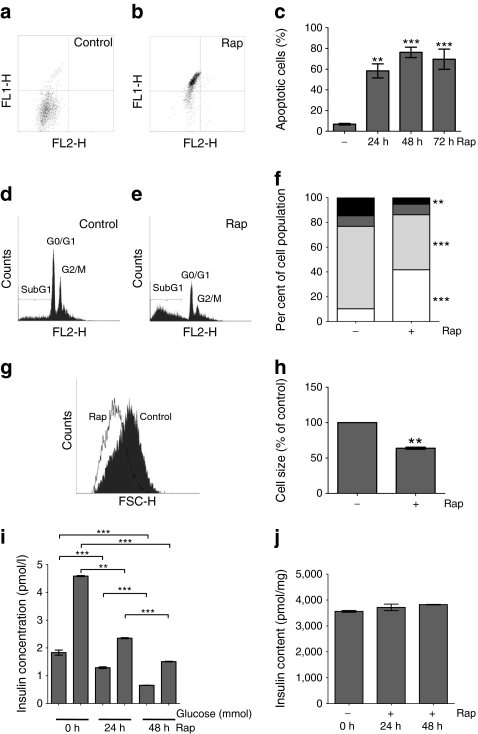
Rapamycin has deleterious effects on MIN6 cell viability and function. MIN6 cells were treated with 200 nmol/l rapamycin as indicated. **a**, **b** Rapamycin decreases viability. Following treatment, cells were dispersed, stained with annexin V and propidium iodide, and then analysed by flow cytometry: *y*-axis (FL1-H), propidium iodide; *x*-axis (FL2-H), annexin V. Representative histograms of control cells (**a**) and cells treated with rapamycin (**b**) for 24 h are shown. **c** Quantification of percentage of apoptotic cells was performed by flow cytometry. *p* values were obtained using a one-way ANOVA with Dunnett’s test. **d**, **e** Representative histograms of cell cycle distribution. MIN6 cells were treated with vehicle (100% ethanol; **d**) or 200 nmol/l rapamycin (**e**) for 24 h, and then analysed by flow cytometry. **f** The percentage of cells in subG_1_, G_0_/G_1_, S and G_2_/M phases related to the total cell population were quantified. *p* values were obtained using a two-way ANOVA with Bonferroni post test. Without rapamycin: white, subG_1_, 10.3 ± 0.6; light grey, G_0_/G_1_, 66.5 ± 1.3; dark grey, S, 8.5 ± 0.3; and black, G_2_/M, 14.9 ± 1.3. With rapamycin: white, subG_1_, 41.8 ± 1.5; light grey, G_0_/G_1_, 44.4 ± 2.1; dark grey, S, 8.5 ± 0.8; and black, G_2_/M, 5.3 ± 0.2. **g** Rapamycin caused a decrease in cell size. The histogram (FSC-H) for the G_0_/G_1_ population shows a left-ward shift in response to rapamycin treatment compared with control, demonstrating that rapamycin causes a decrease in cell size. **h** Quantification of data from (**g**). Data were expressed as percentage of control. *p* values were obtained by paired Student’s *t* test. **i** Rapamycin inhibits GSIS. Following rapamycin treatment, cells were incubated in KRB containing 1 mmol/l glucose for 1 h followed by KRB containing 20 mmol/l glucose for a further 1 h. Supernatant fractions were collected and assayed for insulin concentration using ELISA. *p* values were obtained using a one-way ANOVA followed by Bonferroni post-test. For simplicity, not all statistical significances are shown. **j** Cells from (**i**) were lysed and insulin content determined by ELISA. *p* values were obtained using Dunnett’s test with one-way ANOVA. All data are displayed as means±SE, *n* = 3. **p = 0.001–0.01, ***p < 0.001. Results shown are representative of at least three independent experiments. Rap, rapamycin

To investigate the effects of rapamycin on pancreatic beta cell viability and function, the clonal pancreatic beta cell line MIN6 [19] was incubated with 200 nmol/l rapamycin for up to 72 h (Fig. 1). Flow cytometry of annexin V and propidium iodide stained MIN6 cells demonstrated that rapamycin caused a significant loss of viability by 24 h through an increase in apoptosis rather than necrosis (Fig. 1a–f). In addition, rapamycin caused a decrease in beta cell size (Fig. 1g, h) and a reduction in both basal and glucose-stimulated insulin secretion (GSIS; Fig. 1i). However, there was no significant change in intracellular insulin content, indicating that the effects of rapamycin on GSIS are due to a defect in insulin secretion, rather than in insulin synthesis (Fig. 1j).

### Rapamycin inhibits both mTORC1 and mTORC2 in MIN6 cells and rat- and human-isolated islets of Langerhans

**Fig. 2:**
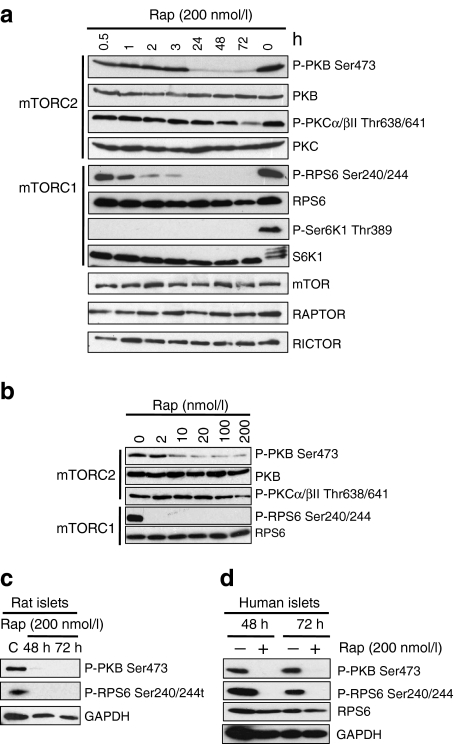
Rapamycin inhibits downstream targets of both mTORC1 and mTORC2. **a** MIN6 cells were treated with 200 nmol/l rapamycin for the time periods indicated. **b** MIN6 cells were treated with 2, 10, 20, 100 and 200 nmol/l rapamycin for 24 h. **c** Rat and (**d)** human islets of Langerhans were treated with 200 nmol/l rapamycin for the times indicated. Following cell lysis, proteins were resolved on SDS-PAGE and immunoblotted using antisera against P-PKB Ser473, P-RPS6 Ser240/Ser244, P-S6K1 Thr389, P-PKCα/βII Thr638/Thr641, as well as total levels of mTOR, RAPTOR, RICTOR, PKB, RPS6, S6K1 and GAPDH. All results are representative of three or, in the case of human islets, two independent experiments. GAPDH, glyceraldehyde-3-phosphate dehydrogenase; Rap, rapamycin

In order to understand the molecular mechanism by which rapamycin is causing beta cell toxicity, the effects of rapamycin on mTOR signalling were investigated. MIN6 cells were treated with rapamycin for up to 72 h and, as expected, rapamycin acutely (within 1 h) inhibited mTORC1 activity as determined by a decrease in the phosphorylation status of S6K on Thr389 and RPS6 on Ser240/244 (Fig. 2a). Interestingly, by 24 h of rapamycin treatment, the phosphorylation of PKB at Ser473, a downstream target of mTORC2 [27], was also significantly inhibited, yet total PKB levels were unaffected (Fig. 2a). Moreover, prolonged rapamycin treatment (48–72 h) also inhibited the turn motif phosphorylation of PKCα and βII on Thr638/641, which is also mediated by mTORC2 [28, 29]. Incubation of MIN6 cells with as little as 10 nmol/l rapamycin for 24 h was sufficient to inhibit both mTORC1 and mTORC2 activity as determined by the phosphorylation state of RPS6 on Ser240/244 and PKB on Ser473 (Fig. 2b). This concentration is similar to that seen in the portal venous system of islet-transplant recipients [30]. These results clearly indicate that rapamycin inhibits both mTORC1 and mTORC2 in MIN6 cells.

Given that MIN6 cells are a clonal beta cell line, it was important to confirm these findings in primary cells. Therefore, isolated rat and human islets of Langerhans were treated with 200 nmol/l rapamycin for 48 and 72 h and the activities of mTORC1 and mTORC2 were determined by assessing the phosphorylation states of RPS6 on Ser240/244 and PKB on Ser473. Rapamycin treatment resulted in the inhibition of both RPS6 on Ser240/244 and PKB on Ser473, indicating that rapamycin also inhibits mTORC1 and mTORC2 in rat (Fig. 2c) and human islets of Langerhans (Fig. 2d). Rapamycin, 200 nmol/l, was used in these experiments and subsequent experiments to produce maximal effects. However, lower doses of rapamycin inhibit mTORC2 and cause cell death in rat islets of Langerhans (see electronic supplementary material [ESM] Fig. 1).

### Rapamycin inhibits mTORC2 through the dissociation of the mTORC2 complex

**Fig. 3:**
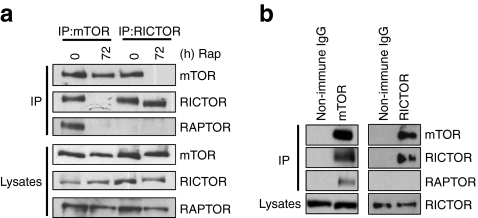
Prolonged rapamycin treatment inhibits mTORC2 activity through dissociation of mTORC2. **a** MIN6 cells were treated with 200 nmol/l rapamycin for 72 h. Cells were lysed in 0.3% CHAPS buffer and immunoprecipitation was performed using anti-mTOR and anti-RICTOR antibodies. **b** Non-immune IgG was used as a control for the immunoprecipitation. Immunoprecipitates and lysates were resolved on SDS-PAGE and immunoblotted using antisera against mTOR, RICTOR and RAPTOR. All results are representative of three independent experiments. IP, immunoprecipitates; Rap, rapamycin

The inhibition of mTORC2 by rapamycin could be via a reduction in the levels of mTORC2 components or the dissociation of the complex. However, treatment of MIN6 cells with rapamycin for up to 72 h caused no reduction in the levels of either mTOR or RICTOR, the principal components of mTORC2 (Figs 2a and 3). Isolation of mTORC1 and mTORC2 complexes by immunoprecipitation with anti-mTOR antibodies followed by analysis revealed that prolonged rapamycin treatment caused the dissociation of both RAPTOR and RICTOR from mTOR, indicating that rapamycin causes the dissociation of both mTORC1 and mTORC2 (Fig. 3). Moreover, isolation of the mTORC2 by immunoprecipitation with anti-RICTOR antibodies followed by analysis of its components confirmed that prolonged rapamycin treatment caused the dissociation of mTOR from RICTOR (Fig. 3). Therefore, rapamycin inhibits mTORC2 in beta cells by causing its dissociation.

### PKB is essential for cell survival and constitutively active PKB protects MIN6 cells and rat islets from the deleterious effects of rapamycin

**Fig. 4:**
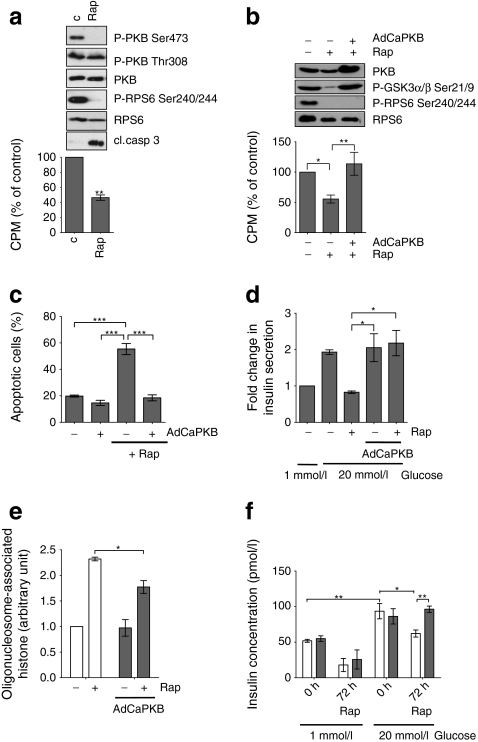
PKB is indispensable for beta cell survival and the overproduction of constitutively active PKB protects MIN6 cells from the deleterious effects of rapamycin. **a**, **b** PKB in vitro activity assay. MIN6 cells were incubated in DMEM supplemented with 15% (vol./vol.) FCS in the absence or presence of rapamycin (200 nmol/l) for 72 h. Endogenous PKB was immunoprecipitated from the lysates and subjected to PKB kinase assay. Lysates previous to PKB kinase assay were collected and separated by SDS-PAGE. Proteins were resolved on SDS-PAGE and immunoblotted using antisera against P-PKB Ser473, P-PKB Thr308, P-RPS6 Ser240/Ser244, P-GSK3α/β Ser21/Ser9, total PKB, RPS6 and cleaved caspase 3. Following cell lysis, PKB was immunoprecipitated using immobilised antibody and kinase activity determined using [γP^32^]ATP and crosstide as the substrate. *p* values were obtained using a one-way ANOVA with Bonferroni post-test. **b** *–* **d** MIN6 cells were mock infected or infected with adenovirus producing constitutively active PKB (AdCaPKB) at a multiplicity of infection of 500 for 24 h, followed by incubation for 72 h (**b**) or 24 h (**c**, **d**) in the presence or absence of 200 nmol/l rapamycin. **c** Following treatment, cells were dispersed, stained with annexin V and propidium iodide, and then analysed by flow cytometry. The percentage of apoptotic cells was quantified. *p* values were obtained using a one-way ANOVA with Bonferroni post-test. **d** Following rapamycin treatment, cells were incubated in KRB containing 1 mmol/l glucose for 1 h, followed by incubation in KRB containing 20 mmol/l glucose for a further 1 h. Supernatant fractions were collected and assayed for insulin concentration using ELISA. Results are expressed as fold change of stimulated insulin secretion over basal insulin secretion. *p* values were obtained using a one-way ANOVA with Bonferroni post-test. All immunoblots are representative of three independent experiments. **e**, **f** Rat islets were infected with adenovirus producing constitutively active PKB for 24 h, followed by a further 48 h incubation in the presence or absence of 200 nmol/l rapamycin. **e** To determine the rate of cell death, internucleosomal DNA fragmentation was analysed. *p* values were obtained using a one-way ANOVA followed by Bonferroni post-test. **f** Following rapamycin treatment, islets were incubated in KRB containing 1 mmol/l glucose for 1 h followed by a further 1 h incubation in KRB containing 20 mmol/l glucose. Supernatant fractions were collected and assayed for insulin concentration using ELISA. *p* values were obtained using a one-way ANOVA followed by Tukey’s multiple comparison test. For simplicity, not all statistical significances are shown. All data are shown as means±SE, *n* = 3. *p = 0.05–0.01, **p = 0.01–0.001 and ***p < 0.001. All immunoblots are representative of three independent experiments. Cl. casp 3, cleaved caspase 3; Rap, rapamycin

PKB is an important pro-survival factor and, as rapamycin causes a decrease in PKB phosphorylation and a decline in cell survival, we further explored the effects of rapamycin on the phosphorylation and activation of PKB. Treatment of MIN6 cells with rapamycin for 24 h resulted in a significant decrease in the PKB phosphorylation at Ser473 but not at Thr308, as well as a significant decrease in its activity (Fig. 4a). This decrease in PKB activity was coincident with an increase in the cleaved form of caspase-3, a cellular marker of apoptosis (Fig. 4a). Moreover, the inhibition of PKB activity results in an increase in apoptosis and a decrease in function in both MIN6 cells and rat islets (ESM Fig. 2).

To determine whether the loss of PKB activity was responsible for rapamycin toxicity, MIN6 cells were infected with an adenovirus producing a myristylated version (constitutively active) of PKB (AdCaPKB), which directs PKB to the plasma membrane and thus confers a constitutively active phenotype [31]. In cells overproducing AdCaPKB, prolonged rapamycin treatment had no detectable effect on PKB kinase activity or on the phosphorylation of the PKB substrate GSK3 (Fig. 4b). Moreover, producing AdCaPKB had no effect on the ability of rapamycin to inhibit the phosphorylation of RPS6 on Ser240/244 and hence mTORC1 (Fig. 4b). Importantly, the production of AdCaPKB in MIN6 cells was found to protect cells from the negative effects of prolonged rapamycin treatment on viability (Fig. 4c) and GSIS (Fig. 4d). To confirm these findings in primary cells, rat islet of Langerhans were infected with AdCaPKB and the effects of rapamycin on islet function and viability were compared with mock-infected islets. Rapamycin treatment for 48 h resulted in increased cell death in both infected and uninfected islets. However, cell death was significantly attenuated in islets infected with AdCaPKB compared with mock-infected islets (Fig. 4e). Rapamycin treatment for 72 h resulted in a significant reduction in GSIS (Fig. 4f). Importantly, islets infected with AdCaPKB were fully protected against the effects of rapamycin on GSIS (Fig. 4f). Therefore, the deleterious effects of rapamycin on beta cell viability and function are likely to be caused by the inhibition of PKB mediated by the inactivation of mTORC2.

### Evidence that inhibition of mTORC2 is primarily responsible for rapamycin toxicity

**Fig. 5:**
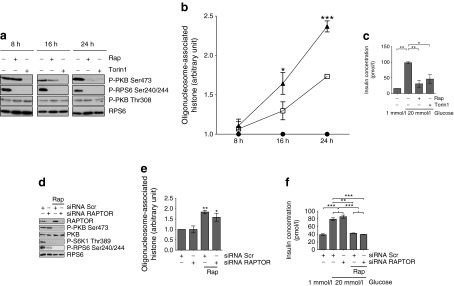
Torin1 causes a reduction in the viability and function of islets of Langerhans. **a** Rat islets were treated with rapamycin (200 nmol/l) or Torin1 (200 nmol/l) for the times indicated. **b** Rat islets were treated as in (a), and internucleosomal DNA fragmentation was determined as an indicator of cell apoptosis using the cell death detection ELISA. *p* values were obtained using a two-way ANOVA followed by Bonferroni post-test comparing rapamycin- and Torin1-treated samples. Black circles, control; white squares, rapamycin; black triangles, Torin1. **c** Rat islets were treated with rapamycin (200 nmol/l) or Torin1 (200 nmol/l) for 40 h, and then incubated in KRB containing 1 mmol/l glucose for 1 h followed by a further 1 h incubation in KRB containing 20 mmol/l glucose. Supernatant fractions were collected and assayed for insulin concentration using ELISA. *p* values were obtained using a one-way ANOVA followed by Bonferroni post-test. **d** Dispersed rat islets were transfected with scrambled or *Raptor* siRNAs for 24 h and then incubated for 48 h in the presence or absence of rapamycin (200 nmol/l). For (**a**) and (**d**), after cell lysis, proteins were separated on SDS-PAGE and western blotted using antisera against RAPTOR, P-PKB Ser473, P-S6K1 Thr389, P-RPS6 Ser240/Ser244, PKB and RPS6. **e** Dispersed rat islets were transfected with scrambled or *Raptor* siRNAs for 24 h prior and then further incubated for 48 h in the presence or absence of rapamycin (200 nmol/l). Apoptosis was determined by measuring using the cell death detection ELISA. *p* values were obtained using two-way ANOVA followed by Bonferroni post-test comparing each column with the first column (siRNA Scr); *n* = 3. **f** Rat islets were transfected and treated as in (**e**), and then incubated in KRB containing 1 mmol/l glucose for 1 h followed by a further 1 h incubation in KRB containing 20 mmol/l glucose. Supernatant fractions were collected and assayed for insulin concentration using an ELISA. *p* values were obtained using one-way ANOVA followed by Tukey’s multiple comparison test, *n* = 3. All data are shown as means±SE. *p = 0.05–0.01, **p = 0.01–0.001 and ***p < 0.001. All immunoblots are representative of three independent experiments. Rap, rapamycin; Scr, scrambled

To provide supportive evidence that mTORC2 plays a critical role in beta cell survival and function, isolated rat islets of Langerhans were treated for up to 24 h with rapamycin or Torin1, a novel selective mTOR inhibitor that, unlike rapamycin, rapidly inhibits both mTORC1 and mTORC2 [18] (Fig. 5). Torin1 had inhibited both mTORC1 and mTORC2 by 8 h as determined by the phosphorylation state of RPS6 and PKB on Ser473, respectively (Fig. 5a). Torin1 or rapamycin had no effect on the phosphorylation of PKB at Thr308. Torin1 also caused a rapid increase in the rate of apoptosis (Fig. 5b), and a decrease in GSIS (Fig. 5c). Compared with islets treated with Torin1, the inhibitory effects of rapamycin on mTORC2 and PKB were delayed (Fig. 5a). Moreover, the rate of cell death was accelerated in Torin1-treated cells compared with rapamycin-treated cells (Fig. 5b). Therefore, it is the inhibition of mTORC2, rather than mTORC1, that correlates with decreased viability and function. To provide further evidence to support these findings mTORC1 function was inhibited in primary rat islets of Langerhans by knocking down *Raptor* expression using siRNA (Fig. 5d–f). This had no significant effect on either islet viability (Fig. 5e) or GSIS (Fig. 5f), yet led to a significant decrease in the phosphorylation of S6K1 and RPS6 (Fig. 5d).

### Downregulation of Rictor expression causes a loss of islet of Langerhans viability

**Fig. 6:**
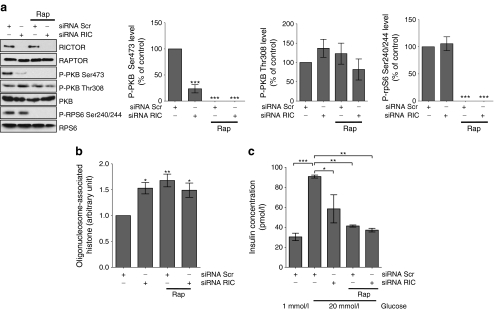
siRNA knockdown of *Rictor* expression causes a loss of beta cell viability. **a** Dispersed rat islets were transfected with scrambled or *Rictor* siRNAs for 24 h and then incubated for 48 h in the presence or absence of rapamycin (200 nmol/l). Cells were then lysed, proteins were resolved on SDS-PAGE and western blotted using antisera against RICTOR, RAPTOR, P-PKB Ser473, P-PKB Thr308, P-RPS6 Ser240/Ser244, PKB and RPS6. Levels of P-PKB Ser473, P-PKB Thr308 and P-RPS6 Ser240/244 were quantified by densitometry and expressed as percentage of control (scrambled siRNA). *p* values were obtained using one-way ANOVA followed by Bonferroni post-test. **b** Dispersed rat islets were transfected with a scrambled siRNA or *Rictor* siRNA for 24 h and then incubated for 48 h in the presence or absence of rapamycin (200 nmol/l). Apoptosis was determined using the cell death detection ELISA (see Methods) following manufacturer’s instructions. *p* values were obtained using two-way ANOVA followed by Bonferroni post-test comparing each column to the first column (siRNA Scr); *n* = 4. **c** Rat islets were transfected and treated as in (a), and then incubated in KRB containing 1 mmol/l glucose for 1 h followed by a further 1 h incubation in KRB containing 20 mmol/l glucose. Supernatant fractions were collected and assayed for insulin concentration using ELISA. *p* values were obtained using one-way ANOVA followed by Tukey’s multiple comparison test, *n* = 3. All data are shown as means±SE. *p = 0.05–0.01, **p = 0.01–0.001 and ***p < 0.001. Results from (**a**) are from five independent experiments; immunoblots from one representative experiment are shown. Results from (**b**) and (**c**) are from three independent experiments. Rap, rapamycin; RIC, RICTOR; Scr, scrambled

As mTORC2 is required for full PKB activation and PKB protects cells against rapamycin toxicity, we aimed to determine whether there was a causal link between the inhibition of mTORC2, the inactivation of PKB and the loss of islet viability. To investigate this, mTORC2 activity was downregulated in dispersed islets of Langerhans by knocking down *Rictor* expression using siRNA. siRNA-mediated knockdown of *Rictor* led to a decrease in the phosphorylation of PKB on Ser473, indicative of decreased mTORC2 activity, without affecting the phosphorylation of RPS6, a marker of mTORC1 activity (Fig. 6a). This correlated with an increase in apoptosis (Fig. 6b) and a decrease in GSIS (Fig. 6c). Importantly, there was no significant change in the phosphorylation of PKB on Thr308 in islets transfected with *Rictor* siRNA (Fig. 6a; see discussion). Taken together, these results indicate that mTORC2 is essential for beta cell viability.

## Discussion

Rapamycin has been used as the primary immunosuppressant in many islet-transplant programmes over the last decade [1]. This choice has been based on the assumption that rapamycin is less toxic to pancreatic beta cells than other immunosuppressants, such as corticosteroids and tacrolimus. However, rapamycin and its analogues can cause deleterious effects on beta cell mass and islet engraftment [3–5], primarily through induction of beta cell apoptosis. Prior to this study, the molecular basis for this toxicity had not been known.

Although rapamycin is known to inhibit mTORC1 in beta cells, we show that prolonged rapamycin treatment is able to abolish the activity of mTORC2 in MIN6 cells, rat and human islets of Langerhans as demonstrated by either a decrease in the phosphorylation of its downstream target PKB on Ser473 (Fig. 2) and/or a reduction in PKB kinase activity (Fig. 4a). Inhibition of PKB using PKB (Akt) inhibitor (AKTi) also results in the loss of cell viability (ESM Fig. 2), whereas the overproduction of constitutively active PKB rescues MIN6 cells or islets from rapamycin-induced apoptosis (Fig. 4). This is not specifically mediated by the recovery of PKB Ser473 phosphorylation but through an increase in overall PKB activity.

Unlike rapamycin, Torin1 and AKTi rapidly inhibit PKB phosphorylation at Ser473, and also lead to a more rapid loss in beta cell viability compared with rapamycin (Fig. 5 and ESM Fig. 2). Moreover, the inactivation of mTORC2 by knockdown of *Rictor* expression in rat islets causes an increase in apoptosis to a similar extent as rapamycin (Fig. 6b), whereas the inactivation of mTORC1 by knockdown of *Raptor* expression had no significant effect on cell viability (Fig. 5d,e). Therefore, we conclude that the maintenance of mTORC2 activity is critical for beta cell survival and that the inhibition of mTORC2 by rapamycin is likely to be responsible for rapamycin toxicity to pancreatic islets. Yet it was recently reported that βRicKO mice, with beta cell-specific ablation of *Rictor*, do not show an increase in beta cell death, although there is a reduction in islet mass and function [17]. One possible explanation for this apparent contradiction is that although PKB phosphorylation at Ser473 is compromised in the islets isolated from βRicKO mice, the phosphorylation of PKB at Thr308, which is mediated by phosphoinositide-dependent kinase 1 (PDK1) [32], is enhanced [17]. This is likely to have a compensatory effect on the activity of PKB. However, the overall kinase activity of PKB in islets from βRicKO mice was not reported by Gu et al. [17]. In contrast, we have been unable to detect any significant increase in the phosphorylation of PKB at Thr308 in rat islets in which *Rictor* expression was acutely knocked down (Fig. 6a) or in islets in which mTORC2 activity was inhibited by either rapamycin or Torin1 (Figs 5a and 6a). Therefore, it is plausible that in βRicKO mice, signalling events downstream of PKB Thr308 phosphorylation rescue beta cells from apoptosis.

PKB integrates upstream survival signals to maintain beta cell viability. It protects beta cells from streptozotocin-induced cell death and mediates the anti-apoptotic actions of insulin, IGF1 and glucagon-like peptide 1 (GLP-1; reviewed in Xie et al. and Elghazi et al. [8, 33]). Of note, mice with knockout of *Pkbβ* (also known as *Akt2*), unlike those with knockout of *Pkbα* (also known as *Akt1*) [34], have a decrease in beta cell mass that parallels an increase in beta cell apoptosis [35]. Interestingly, it has recently been reported that the inhibition of PKB phosphorylation on Ser473 by rapamycin in primary rat and human platelets correlates with a decrease in the activity of PKBβ rather than PKBα [36], Therefore, it is tempting to speculate that rapamycin islet toxicity is caused by the specific impairment of PKBβ activity via the loss of Ser473 phosphorylation caused by the inactivation of mTORC2. Moreover, decreases in PKB phosphorylation in transgenic mice where components of the IRS–PDK1 pathway are ablated (reviewed in Elghazi et al. [33]), or mice producing constitutively active S6K in which IRS signalling is impaired [37], correlate with decreased viability. These anti-apoptotic effects of PKB may be mediated by the nuclear exclusion and degradation of the FOXO family of proteins, which is controlled by the phosphorylation of PKB on Ser473 [38]. However, rapamycin was unable to inhibit the phosphorylation of FOXO (ESM Fig. 3), and mTORC2 ablation in beta cells from βRicKO mice leads to an increase in protein production of FOXO1 and its nuclear retention, yet it does not result in an increase in apoptosis [17]. Therefore, it is likely that the positive role of PKB in beta cell viability is mediated by other downstream targets implicated in cell survival, such as B cell CLL/lymphoma 2 (BCL-2) family members, pro-caspase-9 and murine double minute 2 (MDM2) (reviewed in Hers et al. [39]).

We, in this report, and others have shown that long-term rapamycin treatment inhibits GSIS [3–5]. However, GSIS is not affected by short-term rapamycin treatment [3, 40, 41] and is unaffected by *Raptor* knockdown (Fig. 5f), indicating that the deleterious effects of rapamycin on GSIS are not mediated through the inhibition of mTORC1 but caused by the inhibition of mTORC2. Although mTORC1 regulates protein synthesis, rapamycin has little effect on either insulin synthesis [42] or insulin content (Fig. 1j and [43]) in vitro. Yet the chronic inhibition of mTORC1 in vivo may lead to decreased insulin content which, in turn, could impact on GSIS. However, the inhibition of GSIS by rapamycin has been reported to be caused by reduced mitochondrial ATP production [43].

The findings of this study have important implications for clinical islet transplantation. First, it brings into question the use of rapamycin as a primary immunosuppressant in islet transplantation. However, there are limited alternatives as glucocorticoids and tacrolimus have more significant detrimental effects on beta cells. An alternative is mycophenolate mofetil (MMF); however, studies on human islets have shown that MMF treatment results in a significant reduction in GSIS [41]. The key role of mTORC2 and PKB in beta cell function and survival (this report and others [44–48]) is of importance to those involved in the development of novel immunosuppressive agents for islet transplantation. Ideally, any new agents should not affect PKB activity. One potential area for development is mTORC1-specific inhibitors, which should retain the immunosuppressive effects of rapamycin without any mTORC2-mediated toxicity. However, this makes the assumption that the immunosuppressive effects of rapamycin are indeed mediated solely via mTORC1 rather than mTORC2. In addition, the in vivo activation of PKB might improve the outcome of islet transplantation by improving the function and survival of transplanted beta cells.

In conclusion, we have shown that the molecular basis of rapamycin-induced islet toxicity is through the dissociation and inhibition of mTORC2 and the subsequent reduction in PKB phosphorylation at Ser473 and the suppression of its kinase activity. As a consequence, this work has revealed an important role for mTORC2 in beta cell survival.

## Electronic supplementary material

Below is the link to the electronic supplementary material.

ESM Fig. 1 Dose-dependent effect of rapamycin on rat islet viability. **a** Rat islets of Langerhans were treated with increasing concentrations of rapamycin for 48 h. Cells were lysed and proteins were separated on SDS-PAGE and Western blotted against phosphorylated (P)-PKB Ser473 (S473), P-rpS6 Ser240/Ser244 (S240/244), and total rpS6 as loading control. **b** Rat islets were treated as in **a**, internucleosomal DNA fragmentation was determined as an indicator of cell apoptosis using the Cell Death Detection ELISA. *P* values were obtained using a one-way ANOVA followed by Dunnett’s test. Data are shown as means±SE, *n* = 3. **P = 0.01−0.001, ***P < 0.001 (PDF 32 kb)

ESM Fig. 2 Inhibition of PKB led to an increase in beta-cell death. **a** MIN6 cells were treated with 10 μmol/l AKTi for 8, 16, 24 and 40 h, lysates were collected and separated by SDS-PAGE. **b** MIN6 cells were treated with 10 μmol/l AKTi for 40 h, lysates were collected and subjected to cell death analysis by flow cytometry. *P* values were obtained using a paired student’s *t*-test. **c** Rat islets of Langerhans were treated with 10 μmol/l AKTi for 8 h, lysates were collected and analysed by SDS-PAGE and western blotting using antisera against phosphorylated (P)-PKB Ser473 (S473), (P)-PKB Thr308 (T308), P-rpS6 Ser240/Ser244 (S240/244), and total rpS6 as loading control. **d** Rat islets were treated as in **c** for 8, 16, 24 and 40 h, internucleosomal DNA fragmentation was determined as an indicator of cell apoptosis using the Cell Death Detection ELISA. *P* values were obtained using a two-way ANOVA followed by Bonferroni post-test. **e** Rat islets were incubated in the presence or absence of AKTi for 40 h and insulin secretion assay was performed. *P* values were obtained using a one-way ANOVA followed by Bonferroni post-test. All data are shown as means±SE, *n* = 3. **P = 0.01−0.001, ***P < 0.001 (PDF 38 kb)

ESM Fig. 3 Rapamycin does not inhibit FoxO phosphorylation. MIN6 cells were treated with 200 nmol/l rapamycin for 24, 48 and 72 h, lysates were collected and analysed by SDS-PAGE, followed by western blotting using antisera against phosphorylated (P)-FoxO1/FoxO3a Thr24/Thr32, P-rpS6 Ser240/Ser244 (S240/244), and GAPDH as loading control (PDF 29 kb)

## Abbreviations

AdCaPKB: Adenovirus producing a myristylated version (constitutively active) of PKB
CHAPS: 3-[(3-Cholamidopropyl)dimethylammonio]-1-propanesulfonate hydrate
FOXO: Forkhead box O
GSIS: Glucose-stimulated insulin secretion
GSK3: Glycogen synthetase kinase 3
mTOR: Mammalian target of rapamycin
mTORC1: mTOR complex 1
mTORC2: mTOR complex 2
P-: Phosphorylated
PDK1: Phosphoinositide-dependent kinase 1
PKB: Protein kinase B
PKC: Protein kinase C
RAPTOR: Regulatory-associated protein of target of rapamycin
βRicKO: Beta cell-specific *Rictor* knockout mice
RICTOR: Rapamycin-insensitive companion of target of rapamycin
RPS6: Ribosomal protein S6
S6K: RPS6 kinase
siRNA: Small interfering RNA
