# Supplementary figures and images for: Rapamycin toxicity in MIN6 cells and rat and human islets is mediated by the inhibition of mTOR complex 2 (mTORC2)
Source: Diabetologia. 2012 Feb 8;55(5):1355–65. doi: 10.1007/s00125-012-2475-7 (PMC3328678; doi:10.1007/s00125-012-2475-7)

a

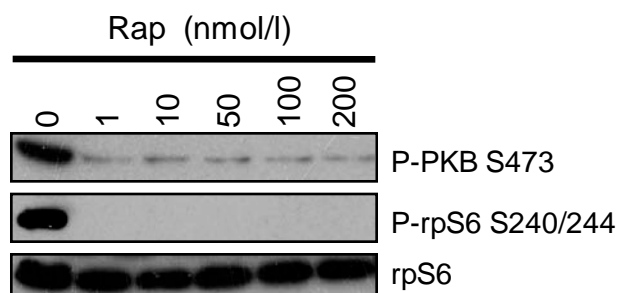

b

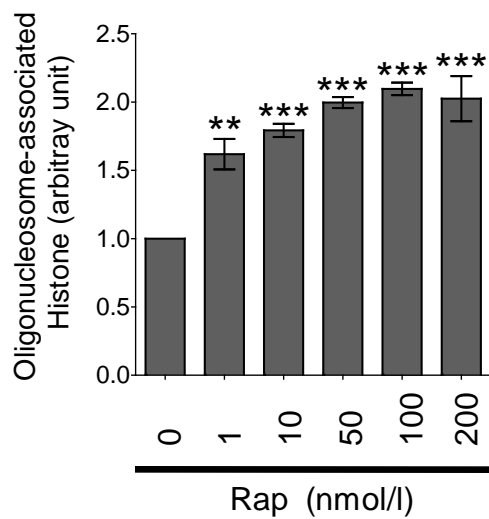

Supplementary Figure 1

Supplement: Supplementary file 1 — Dose-dependent effect of rapamycin on rat islet viability. a Rat islets of Langerhans were treated with increasing concentrations of rapamycin for 48 h. Cells were lysed and proteins were separated on SDS-PAGE and Western blotted against phosphorylated (P)-PKB Ser473 (S473), P-rpS6 Ser240/Ser244 (S240/244), and total rpS6 as loading control. b Rat islets were treated as in a, internucleosomal DNA fragmentation was determined as an indicator of cell apoptosis using the Cell Death Detection ELISA. P values were obtained using a one-way ANOVA followed by Dunnett’s test. Data are shown as means±SE, n = 3. **P = 0.01−0.001, ***P < 0.001 (PDF 32 kb) [file 125_2012_2475_MOESM1_ESM.pdf]

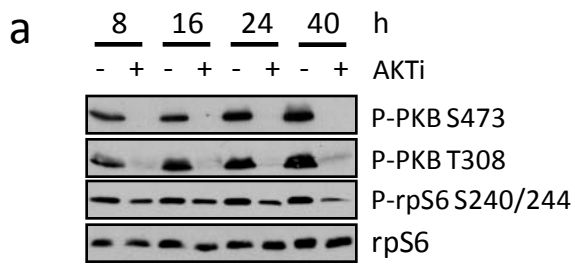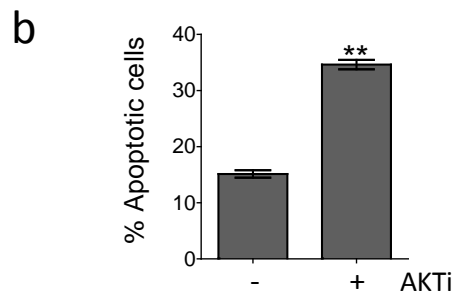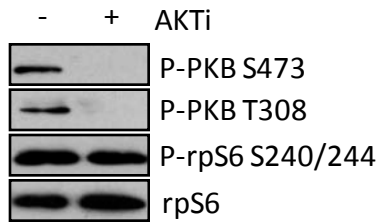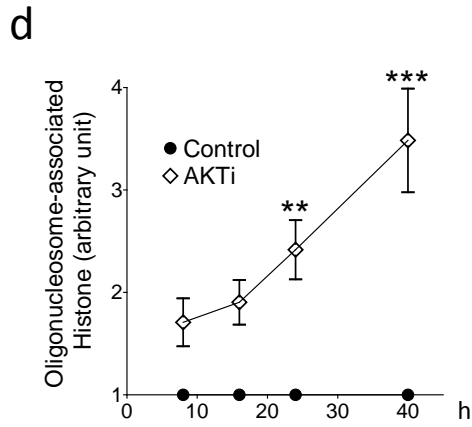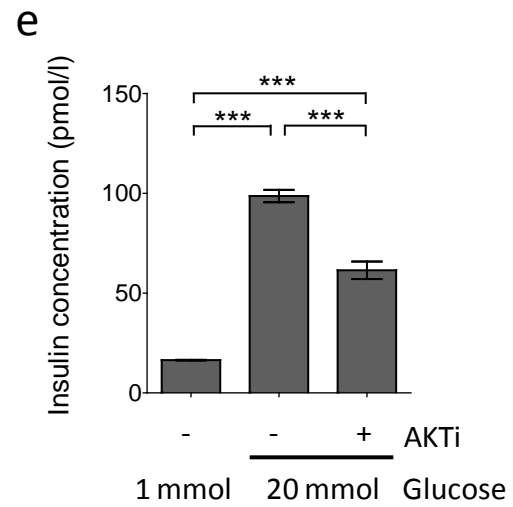

Supplementary Figure 2

Supplement: Supplementary file 2 — Inhibition of PKB led to an increase in beta-cell death. a MIN6 cells were treated with 10 μmol/l AKTi for 8, 16, 24 and 40 h, lysates were collected and separated by SDS-PAGE. b MIN6 cells were treated with 10 μmol/l AKTi for 40 h, lysates were collected and subjected to cell death analysis by flow cytometry. P values were obtained using a paired student’s t-test. c Rat islets of Langerhans were treated with 10 μmol/l AKTi for 8 h, lysates were collected and analysed by SDS-PAGE and western blotting using antisera against phosphorylated (P)-PKB Ser473 (S473), (P)-PKB Thr308 (T308), P-rpS6 Ser240/Ser244 (S240/244), and total rpS6 as loading control. d Rat islets were treated as in c for 8, 16, 24 and 40 h, internucleosomal DNA fragmentation was determined as an indicator of cell apoptosis using the Cell Death Detection ELISA. P values were obtained using a two-way ANOVA followed by Bonferroni post-test. e Rat islets were incubated in the presence or absence of AKTi for 40 h and insulin secretion assay was performed. P values were obtained using a one-way ANOVA followed by Bonferroni post-test. All data are shown as means±SE, n = 3. **P = 0.01−0.001, ***P < 0.001 (PDF 38 kb) [file 125_2012_2475_MOESM2_ESM.pdf]

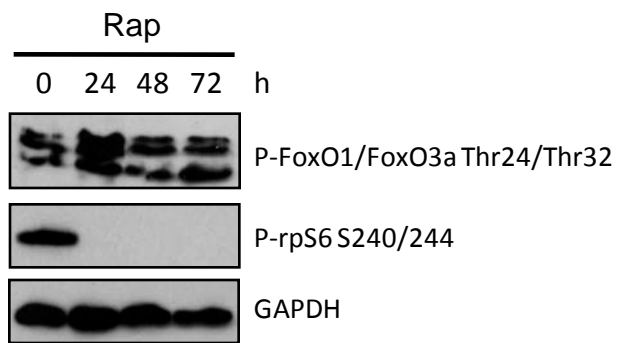

Supplementary Figure 3

Supplement: Supplementary file 3 — Rapamycin does not inhibit FoxO phosphorylation. MIN6 cells were treated with 200 nmol/l rapamycin for 24, 48 and 72 h, lysates were collected and analysed by SDS-PAGE, followed by western blotting using antisera against phosphorylated (P)-FoxO1/FoxO3a Thr24/Thr32, P-rpS6 Ser240/Ser244 (S240/244), and GAPDH as loading control (PDF 29 kb) [file 125_2012_2475_MOESM3_ESM.pdf]
